# Metallic Stents for Proximal Tracheal Stenosis: Is It Worth the Risk?

**DOI:** 10.1155/2012/450304

**Published:** 2012-07-16

**Authors:** Sandeep Bansal, Shruti Dhingra, Babita Ghai, Ashok K. Gupta

**Affiliations:** ^1^Department of Otolaryngology, PGIMER, Chandigarh-160012, India; ^2^Department of Anaesthesia, PGIMER, Chandigarh-160012, India

## Abstract

*Objective*. To demonstrate the risk associated with blocked proximal tracheal stents when a patient presents with acute respiratory distress, with blockage of stent and what is the best management we can offer without damage to the stent and its associated complications. *Case Report*. A 22-yr-old, male patient, presented in severe respiratory distress. He had history of corrosive poisoning for which he was tracheotomised. A stainless steel wire mesh stent was placed in the trachea, from the subglottis, to just above the carina. One month later, he presented with a critically compromised airway with severe respiratory distress. Emergency tracheostomy was done and the metallic stent had to be cut open, in order to provide an airway. *Conclusion*. Management of blocked proximal stents with patient in respiratory distress remains a challenge. Formation of granulation tissue is common and fibreoptic bronchoscopic assisted intubation may not always be possible. A regular follow up of all patients with stents is essential. Placement of stents within a few centimetres of cricotracheal junction should not be encouraged for long term indications.

## 1. Introduction

With advancement in the field of thoracic medicine and development of technology, large numbers of patients are now being treated with tracheal stents. Advancement in stent design and development of both covered and uncovered expandable metallic stents have broadened both indications and durability. As their use has flourished, so have the potential complications associated with them. It is of immense importance for both the ENT surgeons and anaesthetists to be aware of these complications and to be prepared for successfully managing such patients for elective and emergency operations providing a secure airway without compromising the integrity of the airway stent.

Large airway stenting has traditionally been used as palliative treatment for malignant disorders, and now the indications have diversified to include various benign conditions as well. Considering the frequent complication of blockage of tracheal stents, it is questionable whether the use of tracheal stents, especially in proximal trachea, is justified, especially if the patient presents in the emergency with a compromised airway. In such a situation, knowledge of the precise location of the stent and free tracheal segment available for tracheostomy is vital, as the condition of the patient may not permit time for bronchoscopy and bougie-guided placement of the tracheostomy tube. Repeated attempts and failed intubation may lead to various surgical complications, besides damage to the stent. Since the metallic stents are made of stainless steel/titanium mesh, which cannot be cut easily, tracheostomy remains a challenge in these patients.

 In patients who are candidates for resection and reconstruction, only temporary endoscopic palliation should be considered in preparation for surgery to allow adequate stabilization of the inflammatory lesion [1], while reserving stenting for patients with an extremely high surgical risk, or patients refusing surgery, or for patients with a long stenosis that is not amenable to surgical correction. We present a case of one such patient who presented to the emergency with a blocked proximal tracheal stent, and stent damage could not be prevented in order to provide a secure airway.

## 2. Case Report

A 22- year-old male patient presented to our centre in severe respiratory distress. He had history of corrosive poisoning, one year back, and had been admitted in the ICU for 2 weeks, where he was tracheostomised. 3 months later, he still had difficulty in decannulation and developed left vocal cord paralysis with subglottic edema. At that time, videoendoscopy had revealed more than 70% luminal narrowing, 1 cm below the subglottic area. The stenosed segment, from the lower end of cricoid cartilage up to the tracheostoma, involving the first and second tracheal rings, was opened with a midline incision, and fibrotic scarred tissue was removed. A Montgomery T-tube was placed via the tracheostome. 8 months later, the patient again presented with respiratory distress. This time endoscopy revealed complete obliteration of the tracheal lumen by fibrous tissue. A stainless steel wire mesh stent was placed in the trachea, from the subglottis, to just above the carina. One month following the positioning of tracheal stent, on a visit to this part of the country, the patient presented to our centre with a critically compromised airway. As the patient was being prepared for awake fibre optic intubation, he developed severe respiratory distress.

**Figure 1:**
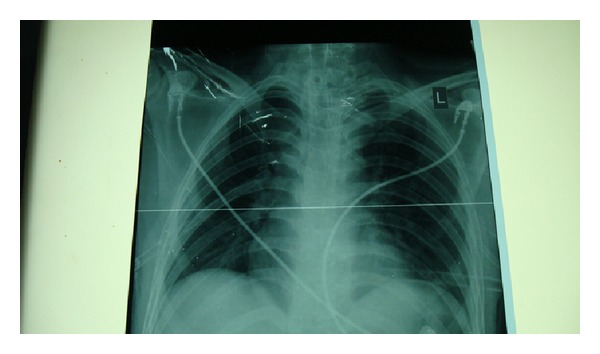
X-ray chest showing tracheostoma in the stent with bilateral chest drains *in situ*.

**Figure 2:**
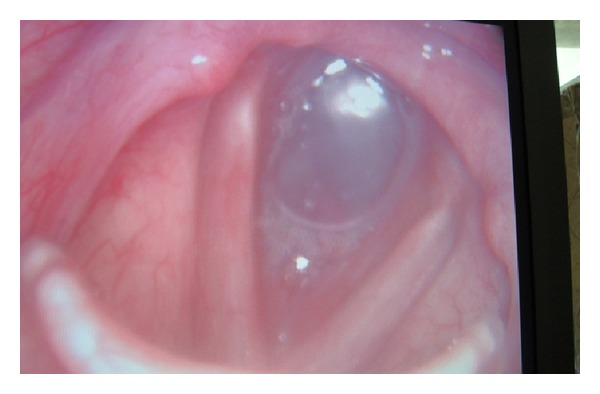
Granulation tissue blocking the proximal trachea from subglottis to the tracheostomy stoma.

Emergency tracheostomy was started, and the metallic stent had to be cut open, in order to provide an airway. The mesh was cut with great difficulty leading to considerable manipulations of the stent. Finally, a cuffed tracheostomy tube of I.D. 6 mm was inserted through the stent, and the patient could be ventilated. In the recovery room, the patient complained of chest pain. A postoperative X-ray chest revealed bilateral pneumothorax with right-sided lung collapse with tracheostoma through the stent (Figure 1). Bilateral chest drains were inserted and kept for 4 days. The patient was discharged one week later with good chest expansion and tracheostomy tube *in situ*. At the time of discharge, fibre optic bronchoscopy done via the tracheostoma revealed proximal tracheal stent lumen blocked completely with granulation tissue (Figure 2).

## 3. Discussion

As demonstrated from the above case report, without endoscopic visualisation, airway manipulation in patients with tracheobronchial stents *in situ* can be catastrophic.

For patients with distal tracheal stents, a tracheostomy tube can be placed above the stent and carefully guided into the lumen of the stent using bronchoscopes. However, the challenges arise in case of proximal or complete tracheal stents, which get blocked by granulation tissue. It is difficult to provide a secure airway in such cases without damage to the stent, especially if the patient presents in severe respiratory distress.

Complications of tracheal stents [2] include granulation tissue formation (27%), restenosis (19%), migration (10%), fracture (8%), erosion (4%), and bleeding (1%). Granulation tissue formation may be mild enough to remain asymptomatic, moderate to produce stridor, or severe enough to present as life-threatening respiratory distress [3].

Covered stents prevent tumour or granulation tissue to proliferate through the stent. However, presence of a covering hampers expectoration of sputum, thus increasing infection. Uncovered stents, though not increasing the risk of respiratory infection, do allow granulation tissue to proliferate within the stent, thus blocking its lumen and causing difficulty in stent removal.

Metallic stents are more prone to develop granulations [4] especially at the proximal segment, as they are more rigid with multiple edges and produce circumferential pressure on tissues, leading to airway irritation.

The subglottic airway, with its intact cricoid ring, is not distensible, unlike the proximal trachea. The cricotracheal junction is therefore subjected to greater degrees of motion with head movement [2]. The excessive shearing forces prevent stent mucosalization and lead to granulation tissue formation, stent fracture, and restenosis around the stent orifice. This granulation tissue that forms around metal stents also leads to epithelialization of the stent making its removal extremely difficult. Once the stents become mucosalised, they may require destruction and piecemeal removal [4] as early as one month.

It is suggested that in patients with tracheobronchial stents, rigid and fibreoptic bronchoscopy should be used if percutaneous tracheostomy is indicated so as to guide the tracheostomy tube through the stent and avoid perforation or dissection of the trachea and formation of a false passage with minimal stent damage. Also, a regular followup is essential to prevent complications from occurring, especially formation of granulation tissue, which may completely block the stent, and does not provide adequate time to the surgeon or anaesthetist to establish a secure airway. Moreover, despite the relative simplicity of stenting techniques, in light of the potential complications suggested, this technique should not be used as a long-term solution for problems in the proximal airway, especially when the stent is placed adjacent to (within 1 cm) the cricotracheal junction.

The optimal treatment of tracheal stenosis remains undefined. Traditionally, tracheal stenosis has been managed by thoracic surgeons and otolaryngologists. Endoscopic procedures are usually performed as a bridge to definitive surgical intervention. Tracheal resection and anastomosis is now accepted as the procedure of choice for tracheal stenosis, with excellent results [5–11]. Various different, complex, and technically challenging surgical techniques have been described when subglottis is involved with proximal trachea from very experienced groups obtaining excellent results [12–14].

Laser therapy is now recommended only in patients with true contraindications to surgery [15]. In patients who are unfit for surgery (high-risk patients, patients refusing surgical options), tracheal stents are a good option [16, 17]. Recently, studies have been initiated to investigate treatment options (a tissue-engineered airway, revascularized allografts in the heterotopic position, and cryopreserved aortic allografts) for extensive lesions in postintubation tracheal stenosis [18–20].

## References

[1] Wood DE (2001). Bronchoscopic preparation for airway resection. *Chest Surgery Clinics of North America*.

[2] Burningham AR, Wax MK, Everts EC, Andersen PE, Cohen JI (2002). Metallic tracheal stents: complications associated with long-term use in the upper airway. *Annals of Otology, Rhinology and Laryngology*.

[3] Filler RM, Forte V, Chait P (1998). Tracheobronchial stenting for the treatment of airway obstruction. *Journal of Pediatric Surgery*.

[4] Zakaluzny SA, Lane JD, Mair EA (2003). Complications of tracheobronchial airway stents. *Otolaryngology*.

[5] Couraud L, Jougon JB, Velly JF (1995). Surgical treatment of nontumoral stenoses of the upper airway. *Annals of Thoracic Surgery*.

[6] Grillo HC, Donahue DM, Mathisen DJ, Wain JC, Wright CD (1995). Postintubation tracheal stenosis: treatment and results. *Journal of Thoracic and Cardiovascular Surgery*.

[7] París F, Borro JM, Tarrazona V (1990). Management of non-tumoral tracheal stenosis in 112 patients. *European Journal of Cardio-Thoracic Surgery*.

[8] Wright CD, Grillo HC, Wain JC (2004). Anastomotic complications after tracheal resection: Prognostic factors and management. *Journal of Thoracic and Cardiovascular Surgery*.

[9] Grillo HC, Mathisen DJ, Wain JC (1992). Laryngotracheal resection and reconstruction for subglottic stenosis. *Annals of Thoracic Surgery*.

[10] Jaquet Y, Lang F, Pilloud R, Savary M, Monnier P (2005). Partial cricotracheal resection for pediatric subglottic stenosis: long-term outcome in 57 patients. *Journal of Thoracic and Cardiovascular Surgery*.

[11] Rea F, Callegaro D, Loy M (2002). Benign tracheal and laryngotracheal stenosis: surgical treatment and results. *European Journal of Cardio-thoracic Surgery*.

[12] Monnier P, Lang F, Savary M (2003). Partial cricotracheal resection for pediatric subglottic stenosis: a single institution's experience in 60 cases. *European Archives of Oto-Rhino-Laryngology*.

[13] Pearson FG, Cooper JD, Nelems JM, Van NostrandA. W.P. WP (1975). Primary tracheal anastomosis after resection of the cricoid cartilage with preservation of recurrent laryngeal nerves. *Journal of Thoracic and Cardiovascular Surgery*.

[14] Terra RM, Minamoto H, Carneiro F, Pego-Fernandes PM, Jatene FB (2009). Laryngeal split and rib cartilage interpositional grafting: treatment option for glottic/subglottic stenosis in adults. *Journal of Thoracic and Cardiovascular Surgery*.

[15] Rea F, Callegaro D, Loy M (2002). Benign tracheal and laryngotracheal stenosis: surgical treatment and results. *European Journal of Cardio-thoracic Surgery*.

[16] Isa AY, Macandie C, Irvine BW (2006). Nitinol stents in the treatment of benign proximal tracheal stenosis or tracheomalacia. *Journal of Laryngology and Otology*.

[17] Majid A, Fernandez-Bussy S, Kent M (2012). External fixation of proximal tracheal airway stents: a Modified technique. *The Annals of Thoracic Surgery*.

[18] Macchiarini P, Jungebluth P, Go T (2008). Clinical transplantation of a tissue-engineered airway. *The Lancet*.

[19] Delaere P, Vranckx J, Verleden G, De Leyn P, Van Raemdonck D (2010). Tracheal allotransplantation after withdrawal of immunosuppressive therapy. *New England Journal of Medicine*.

[20] Martinod E, Radu DM, Chouahnia K (2011). Human transplantation of a biologic airway substitute in conservative lung cancer surgery. *Annals of Thoracic Surgery*.

